# Binding of Harmine Derivatives to DNA: A Spectroscopic Investigation

**DOI:** 10.3390/molecules22111831

**Published:** 2017-10-27

**Authors:** Bruno Pagano, Marco Caterino, Rosanna Filosa, Concetta Giancola

**Affiliations:** 1 Department of Pharmacy, University of Naples Federico II, Via D. Montesano 49, 80131 Naples, Italy; bruno.pagano@unina.it (B.P.); marco.caterino@unina.it (M.C.); 2 Department of Experimental Medicine, Campania University “Luigi Vanvitelli”, Via L. De Crecchio 7, 80138 Naples, Italy; rosanna.filosa@unicampania.it

**Keywords:** alkaloids, DNA duplex, spectroscopy

## Abstract

Harmine belongs to a group of β-carboline alkaloids endowed with antitumor properties. Harmine and its derivatives are thought to bind to DNA and interfere with topoisomerase activities. We investigated the base-dependent binding of harmine, and three of its synthetic anticancer-active derivatives to the genomic DNA from calf thymus and two synthetic 20-mer double helices, the poly(dG-dC)·poly(dG-dC) and the poly(dA-dT)·poly(dA-dT), by means of UV-Vis and circular dichroism (CD) spectroscopies. The data show that the DNA binding and stabilising properties of the investigated derivatives are base pair-dependent. These results could be used as a guide to design and develop further bioactive analogues.

## 1. Introduction

Harmine (HR) is a widespread natural tricyclic β-carboline alkaloid, isolated from the seeds of *Peganum harmala*, a middle-eastern plant that has been known about for a long time and used in folk medicine [1,2,3]. Its structure is characterised by a pyridine ring fused to an indolyl ring, a methyl and a methoxy group at positions 1 and 7, respectively. Regardless of popular beliefs, there is a kernel of truth in the supposed beneficial effects of HR as it was proved to possess many very interesting properties, including antimicrobial, antioxidative, antitumor, anti-inflammatory, cytotoxic activity, and more [4,5,6,7,8,9,10,11,12]. It goes without saying that the anticancer property has emerged as the foremost alluring facet, prompting many groups to dedicate their efforts to synthesising harmine derivatives with enhanced antitumor properties against human cancer-cells [11,13,14].

The HR antiproliferative mechanism of action is far from being fully disclosed but it has become clear that the DNA intercalation is a driving force. HR is mainly known to interfere with the DNA topoisomerase activity [15], but also to induce DNA damage [16], and to inhibit the telomerase that leads to cell senescence [17]. The key factors are the HR tricyclic planar molecular geometry and identities and arrangement of its substituents [14,18,19].

**Figure 1:**
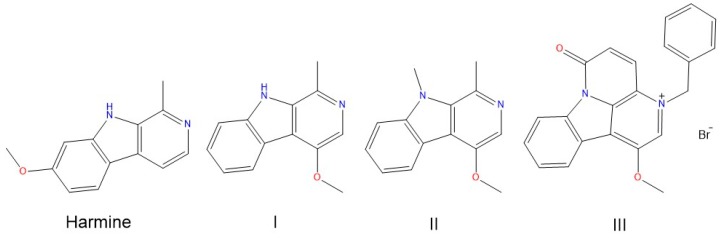
Chemical structure of harmine (7-methoxy-1-methyl-9*H*-pyrido(3,4-*b*)indole), and its synthetic derivatives: **I** (4-methoxy-1-methyl-9*H*-pyrido(3,4-*b*)indole), **II** (4-methoxy-1,9-dimethyl-9*H*-pyrido(3,4-*b*)indole), and **III** (3-benzyl-1-methoxy-6-oxo-6*H*-indole[3,2,1-de]-[1,5]-naphthyridinin-3-ium bromide).

To achieve a deeper understanding of the HR-derivatives binding to the DNA, herein we report a UV/Vis- and CD-based investigation of the DNA-binding properties of three synthetic HR derivatives (compounds **I**–**III**, Figure 1), which were previously proved to have enhanced activity against human prostate cancer cells (PC-3) in the µM range [14]. The joint UV/Vis-circular dichroism approach is widely adopted for studying DNA-ligand systems [18,20,21]. It is readily diagnostic of the ligand-DNA interaction and of the ensuing stabilisation effects, as well as for the binding-induced structural perturbations. Last and by no means least, the required sample amount is modest compared to other techniques. Calf thymus DNA (ctDNA) was used as a reference, and two synthetic 20-mer duplexes were investigated for assessing the differential behaviour against GC and AT segments: the poly(dG-dC)·poly(dG-dC), hereafter d(GC)_10_, and the poly(dA-dT)·poly(dA-dT), referred to as d(AT)_10_ from now on.

On a structural basis, **I** differs from HR in the methoxy group at position 4 rather than 7. Compound **II** has an additional methyl group at position 9 with respect to **I**, whereas **III** features a fourth ring fused to the β-carboline structure, with a methoxy in position 4 and a benzyl group bound to the pyridine nitrogen (Figure 1).

## 2. Results

### 2.1. UV-Vis

**Figure 2:**
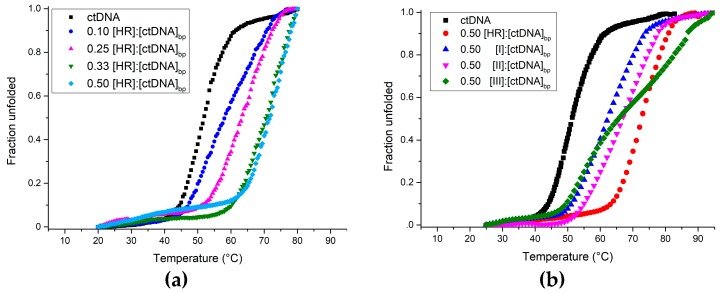
Normalised UV-Vis thermal melting profiles of *Calf thymus* DNA (ctDNA) at increasing [HR]:[ctDNA]_bp_ ratio (**a**); and at [ligand]:[ctDNA]_bp_ of 0.50 (**b**). The ligand concentrations were increased over a fixed (ctDNA)_bp_. Melting curves were obtained monitoring the absorbance at 260 nm as a function of temperature.

The interaction between the investigated compounds and the DNA was analysed by UV-Vis spectroscopy. First, UV melting curves of the ctDNA at increasing HR concentration were recorded to evaluate the HR binding and stabilising capability. Harmine sizeably stabilises the ctDNA even at the 0.10 [harmine]:[ctDNA]_bp_ ratio, whereas only a slight difference occurs between the UV thermal denaturation profiles at [harmine]:[ctDNA]_bp_ ratios of 0.33 and 0.50, with the latter showing the maximum stabilizing effect (Δ*T*_m_ = 20 °C) (Figure 2a). The 0.50 ratio was thereby picked as a standard for the measures to follow, for studying the interaction between HR and its synthetic derivatives with either ctDNA, d(GC)_10_ or d(AT)_10_.

As Figure 2b depicts and Table 1 summarises, UV melting data clearly show that each of the ligands stabilises the ctDNA [22]. Harmine works better than **I** and **II**, although, interestingly, the melting profile of the ctDNA with **III** exhibits two inflection points.

**Figure 3:**
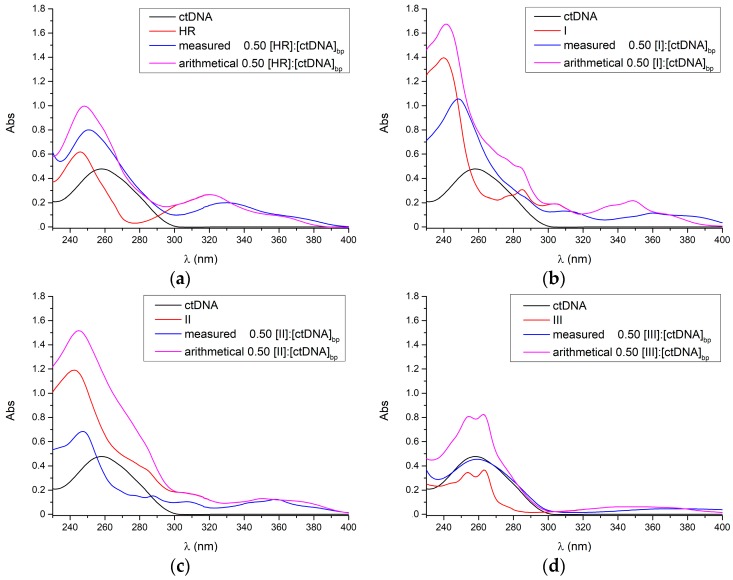
UV absorption spectra of the ctDNA with (**a**) harmine; (**b**) **I**; (**c**) **II**; and (**d**) **III**. The arithmetical ligand-ctDNA sum spectra are reported as well.

Figure 3 shows the UV-Vis spectra of both the free and ctDNA-bound ligands, which all intensely absorb in the 240–300 nm region and lesser in the 300–400 nm range. Each experimental ligand-ctDNA spectrum sizeably differs from the corresponding arithmetical ligand-ctDNA sum, highlighting the ligand-ctDNA interaction.

**Figure 4:**
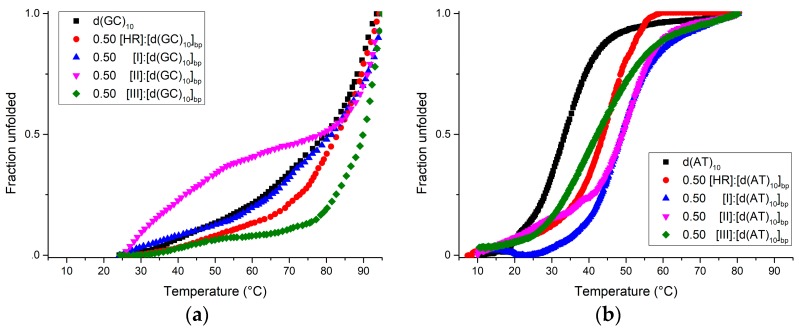
Normalised UV thermal denaturation curves of d(GC)_10_ (**a**) and d(AT)_10_ (**b**) in the absence and presence of ligands at 0.50 [ligand]:[DNA]_bp_ ratio followed at 260 nm.

Further base-dependent information came from the melting experiments performed with either d(GC)_10_ (Figure 4a) or d(AT)_10_ (Figure 4b). The melting temperatures in the case of d(AT)_10_ complexes are well detected. On the contrary, the *T*_m_ values of the d(GC)_10_ complexes are invariably higher than 80 °C, but not accurately measured due to the upper heating limit both in the absence and in the presence of the ligands, as already reported in the case with GC sequences in similar experimental conditions [20,21]. In any case, **III** clearly makes the most stable complex with the d(GC)_10_, bearing witness to the key role that the benzyl group at position 3 and/or the fourth ring have in the interaction with GC-rich segments (Figure 3a and Table 1). In the case of d(AT)_10_ (Figure 4b), the utmost stabilisation was achieved with both **I** and **II**, whereas **III** and HR affect the *T*_m_ to a smaller extent.

### 2.2. Circular Dichroism

**Figure 5:**
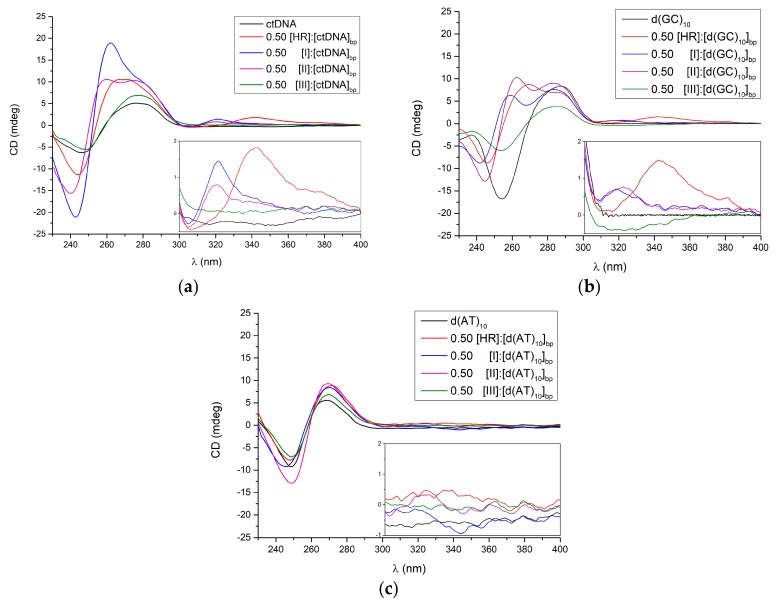
Circular dichroism spectra of (**a**) ctDNA; (**b**) d(GC)_10_; and (**c**) d(AT)_10_, either free or as bound at 0.50 [ligand]:[DNA]_bp_ ratio to the harmine or its three derivatives.

Circular dichroism (CD) spectroscopy was also employed for gaining insights into the conformational changes occurring upon β-carboline alkaloids binding to the DNA. Figure 5a shows the CD spectra of the ligand-free and ligand-bound ctDNA ([ligand]:[ctDNA]_bp_). The addition of HR, **I** or **II** extensively affects the ctDNA CD spectrum in the 240–300 nm interval, while minor changes occur upon the administration of **III**. New induced dichroic signals (iCD) arise in the 300–400 nm region purely due to the complex formation, since neither the DNA nor any of the ligands absorb in this range. The iCDs arise in the 300–400 nm range for all the complexes except for the one involving **III**.

The iCD finds root in the transition moments coupling of both the ligand and the surrounding environs by the DNA, that is to say the reciprocal molecular orientation. While the iCD magnitude can hardly be accounted for, its sign relies strongly on the orientation of the ligand with respect to the DNA at the binding site [23,24]. This means the ligands can intercalate along the DNA axis as they are all oriented in parallel planes or alternately flipped each other. The former case prompts a net CD signal as seen for compounds **I** and **II**, whereas the latter might be the case with compound **III** where positive and negative contributions elide each other.

The binding effects of HR, **I**, **II**, and **III** on the CD spectra of d(GC)_10_ and d(AT)_10_ are shown in Figure 5b,c. The CD spectrum of the d(GC)_10_ is markedly affected by all the ligands and, again, iCDs in the 300–400 nm region appear, with the exception of the complex made by **III**.

The CD spectrum of d(AT)_10_ is instead slightly modified by each of the ligands in the 240–340 nm range yet no iCDs are detected in this case (Figure 5c).

## 3. Discussion

The harmine-derivatives family is very alluring for many purposes, including cancer treatment. The comprehension of the mechanism of action, however, still has blind spots, although the intercalation into DNA duplex is known to be responsible for hampering the DNA replication. Synthetic harmine derivatives were proved to have enhanced antitumor properties according to their molecular geometry and substituents configuration. Little is known about the sequence-specific interaction of these compounds with the DNA and harmine was only recently demonstrated to preferentially bind GC-rich segments [25].

We investigated the interaction of calf thymus DNA and of the d(GC)_10_ and d(AT)_10_ 20-mer duplexes with both harmine and three of its derivatives (compounds **I**–**III**), which recently were proved to have enhanced anticancer activity [14].

UV-Vis absorption confirms all the ligands to stabilise the ctDNA sequence in the order HR > **II** > **I** (Table 1). Sequence-specific behaviours were found as the d(AT)_10_ is greatly stabilised by both **I** and **II** (∆*T*_m_ = +17 °C) and to a smaller extent by HR and **III** (∆*T*_m_ = +10 and +7 °C, respectively). In contrast, compound **III** clearly makes the most stable d(GC)_10_ complex though the upper heating limit, which prevented us from pinpointing the *T*_m_ values. This is consistent with previous findings in resembling experimental conditions [20,21]. In contrast, **I** and **II** barely affect the *T*_m_ of the GC-rich fragment. Notably, the UV thermal denaturation profile of ctDNA in the presence of compound **III** exhibits two inflection points. The more complex ctDNA thermal denaturation process as compared to monotonous 20-mer sequences can make this intelligible. The ctDNA features both AT- and GC-rich shuffled segments that undergo differential thermal denaturation. In terms of *T*_m_, compound **III** has a much weaker effect on AT segments countered by the very strong impact on the GC stretches thereby introducing a massive *T*_m_ gap between the two, which detectable by our UV investigation. In contrast, the much smaller behaviour bias that HR, **I**, and **II** exhibit towards AT and GC results in narrower *T*_m_ splitting, which is conceivably undetectable in a UV melting study, at least in these experimental conditions. Comparable two-inflection UV thermal profile had been reported for a 9-benzyl-substituted harmine derivative compound [18].

Given these findings, cautious structural consideration can be drawn. Although precise Δ*T*_m_ are not available for the d(GC)_10_, the methoxy group position switch from 7 to 4 appears to hinder the d(GC)_10_ stability while sensibly hardening the d(AT)_10_ at a comparison of the melting profiles of HR and **I** reported in Figure 4a,b. No stabilising effect to d(AT)_10_ is appreciable upon addition of the methyl group at position 9, whereas the d(GC)_10_ thermal profile is perturbed. Anyway, one could hypothesise a DNA pre-melting secondary structure transition as already seen for poly(dA-dT) poly(dA-dT) duplex [26,27]. As compared to harmine, the fourth ring with an extra H-bond acceptor, the benzyl group, and the static positive charge featured by **III** markedly enhance the d(GC)_10_-selective thermal stabilisation whilst even bearing reduced effectiveness on d(AT)_10_. 

Among the harmine derivatives previously addressed, compound **III** demonstrated the utmost antiproliferative capacity towards PC-3 cells [14]. Notably, many DNA-intercalating, anticancer-active drugs have indeed been reported to exhibit preferential binding to GC-rich segments, amid a long-lasting hypothesis of a positive correlation between in vivo antitumor activity and the preferential binding to GC-rich DNA sequences [28]. Given these findings and structural observations, **III** may serve as a hit-compound to start from for advancing the rational design of sequence-specific, DNA-intercalating, and anticancer-active small molecules.

## 4. Materials and Methods

### 4.1. Chemicals

The 4-methoxy-1-methyl-9*H*-pyrido(3,4-*b*)indole (**I**), the 4-methoxy-1,9-dimethyl-9*H*-pyrido(3,4-*b*)indole (**II**) and, the 3-benzyl-1-methoxy-6-oxo-6*H*-indole(3,2,1-de)-(1,5)-naphthyridinin-3-ium (**III**) were synthesised according to procedures reported elsewhere [14]. Harmine and its derivatives were dissolved in DMSO. The calf thymus DNA and the 20-mer oligonucleotides were purchased from Sigma Aldrich (Milan, Italy) and biomers.net (biomers.net GmbH, Ulm, Germany), respectively. A 1 mM sodium phosphate, 0.1 mM EDTA buffer at pH 7.0 was used for dissolving the ctDNA and the d(GC)_10_, whereas a 40.0 mM sodium phosphate, 0.1 mM EDTA buffer at pH 7.0 was used for the d(AT)_10_. The ctDNA concentration was determined spectrophotometrically by using the molar extinction coefficient value (λ 260 nm) *per* base-pairs of 13,200 cm^−1^ M^−1^ [29]. The 20-mer d(GC)_10_ and d(AT)_10_ duplex concentrations were determined by the nearest-neighbour method using molar extinction coefficient values at λ 260 nm of 315,988 and 316,144 cm^−1^ M^−1^, respectively [30]. The per base-pair concentrations for the d(AT)_10_ and d(GC)_10_ were calculated by multiplying by twentyfold those of the respective duplex. MilliQ filtered water was used.

### 4.2. UV Measurements

UV-Vis absorption spectra were recorded by using a Jasco V-530 spectrometer (Jasco International Co., Tokyo, Japan) equipped with a PTC-348WI thermoelectrically controlled cell holder (Jasco International Co., Tokyo, Japan), at 10 °C for the d(AT)_10_ and 25 °C for ctDNA and d(GC)_10_ in the 400–230 nm range using twin quartz cells, 0.1 cm optical path. UV melting experiments were registered at 260 nm in the 25–95 °C range for the d(GC)_10_ and 10–80 °C for the d(AT)_10_, temperature ramp 1 °C min^−1^. The UV melting curves were normalised to the 0–1 range and melting temperatures calculated by first derivative.

### 4.3. CD Measurements

A Jasco J-715 spectropolarimeter (Jasco International Co., Tokyo, Japan) was used for circular dichroism measures by using a 0.1 cm path length cuvette in a Peltier-thermostatted cell holder at both 10 and 25 °C. The scan rate was 20 nm min^−1^ and bandwidth 2.0 nm. Spectra were collected in the 400–230 nm range and averaged over three collections. 

## Figures and Tables

**Table 1:** Melting temperatures of the ctDNA, d(GC)_10_, and d(AT)_10_ in the absence and presence of harmine, **I**, **II**, and **III** at 0.50 [ligand]:[DNA]_bp_ ratio obtained by UV melting experiments.

Ligand	*T*_m_ (°C ± 1)		
	ctDNA	d(GC)_10_	d(AT)_10_
No ligand	55	>85	35
Harmine	75	>85	45
**I**	62	>85	52
**II**	65	>85	52
**III**	≈60–≈82	>90	42
